# Local Calcium Elevation and Cell Elongation Initiate Guided Motility in Electrically Stimulated Osteoblast-Like Cells

**DOI:** 10.1371/journal.pone.0006131

**Published:** 2009-07-03

**Authors:** Nurdan Özkucur, Thomas K. Monsees, Srikanth Perike, Hoa Quynh Do, Richard H. W. Funk

**Affiliations:** 1 Department of Anatomy, Medical Faculty Carl Gustav Carus, TU-Dresden, Dresden, Germany; 2 Department of Medical Biosciences, University of the Western Cape, Bellville, South Africa; McMaster University, Canada

## Abstract

**Background:**

Investigation of the mechanisms of guided cell migration can contribute to our understanding of many crucial biological processes, such as development and regeneration. Endogenous and exogenous direct current electric fields (dcEF) are known to induce directional cell migration, however the initial cellular responses to electrical stimulation are poorly understood. Ion fluxes, besides regulating intracellular homeostasis, have been implicated in many biological events, including regeneration. Therefore understanding intracellular ion kinetics during EF-directed cell migration can provide useful information for development and regeneration.

**Methodology/Principal Findings:**

We analyzed the initial events during migration of two osteogenic cell types, rat calvarial and human SaOS-2 cells, exposed to strong (10–15 V/cm) and weak (≤5 V/cm) dcEFs. Cell elongation and perpendicular orientation to the EF vector occurred in a time- and voltage-dependent manner. Calvarial osteoblasts migrated to the cathode as they formed new filopodia or lamellipodia and reorganized their cytoskeleton on the cathodal side. SaOS-2 cells showed similar responses except towards the anode. Strong dcEFs triggered a rapid increase in intracellular calcium levels, whereas a steady state level of intracellular calcium was observed in weaker fields. Interestingly, we found that dcEF-induced intracellular calcium elevation was initiated with a local rise on opposite sides in calvarial and SaOS-2 cells, which may explain their preferred directionality. In calcium-free conditions, dcEFs induced neither intracellular calcium elevation nor directed migration, indicating an important role for calcium ions. Blocking studies using cadmium chloride revealed that voltage-gated calcium channels (VGCCs) are involved in dcEF-induced intracellular calcium elevation.

**Conclusion/Significance:**

Taken together, these data form a time scale of the morphological and physiological rearrangements underlying EF-guided migration of osteoblast-like cell types and reveal a requirement for calcium in these reactions. We show for the first time here that dcEFs trigger different patterns of intracellular calcium elevation and positional shifting in osteogenic cell types that migrate in opposite directions.

## Introduction

Guided cell migration is essential for embryonic development, tissue formation, inflammation and wound-healing [1]–[3]. In addition to chemical (chemotaxis) or mechanical (contact) mechanisms, guided cell migration is influenced by electric potential (electrotaxis) [4]–[7]. Exogenous dcEFs of physiological strength induce a variety of cellular responses [8]–[10]. Although the general effects of electrical stimulation on various cell types are well known, the exact cascades translating exogenous and endogenous electrical signals into a variety of intracellular responses are still poorly understood. One important candidate for this translation is calcium. Calcium is involved in maintaining cell physiology and is a pluripotent signaling molecule that holds a crucial place in many cell biological pathways [11]–[13]. In particular, elevation of cytoplasmic calcium serves as a rapid response to various factors including electrical stimulation. Thus, regulation of intracellular calcium ([Ca^2+^]_i_) levels via non-invasive electrical stimulation may be important for controlling cellular responses during migration [14]. To address this issue here, we observe cell morphological rearrangements and [Ca^2+^]_i_ dynamics in response to dcEFs.

In this study we use two osteogenic cell types, rat calvarial and human SaOS-2 cells [15], [16]. These are good models for directional migration studies since they prefer opposite movement directions in response to electrical stimulation. Immunofluorescence, vital staining, differential interference contrast (DIC) microscopy and time-lapse video microscopy techniques were used to analyze the short- and long-term cellular responses to electrostimulation. We offer novel observations of the initial cellular response to electrical stimulation, in terms of cytoskeletal reorganization and ion fluxes, and suggest an early role for intracellular calcium in directed cell migration.

## Results

### DcEF-induced elongation and reorientation are time- and voltage-dependent

**Figure 1:**
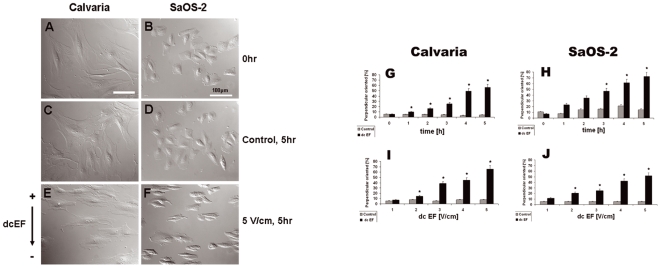
Time- and voltage-dependent cell shape alteration of osteoblast-like cells in a dcEF. DIC micrographs showing perpendicular orientation and elongation of calvaria (A,C,E) and SaOS-2 (B,D,F) cells before (A,B) and after (E,F) exposure to a dcEF of 5 V/cm for 5 h. Control cells (C,D; 5 h, no dcEF) displayed no major alteration in shape or orientation. Graphs indicating the percentage of perpendicular-orientated cells with or without electric stimulation as a function of time (G,H, at 5 V/cm) or voltage (I,J, after 5 hours). Means and standard deviations are shown. Numbers of cells scored: 1000–1500 in total for each time or voltage level. * indicates P<0.05.

We first observed the cell morphological changes induced by exposure to low and high voltage dcEFs for different durations. The field strengths used are consistent with those applied in similar studies on osteoblast cells [17]–[19]. After 5 h of exposure to a 5 V/cm dcEF, most of the calvarial and SaOS-2 cells elongated and orientated perpendicular to the field vector (Fig. 1E and F), in contrast to the control cells (Fig. 1A, B, C and D). Overall, the percentage of perpendicular-orientated calvarial osteoblasts significantly increased with the duration of dcEF stimulation, from 5±0.8% (Mean±standard deviation, start) to 56±2.2% (Mean±standard deviation, 5 h, Fig. 1G). The lower percentage of orientation to start was due to some cells remained round, did not have a significantly elongated axis and hence, were difficult to score. Increasing numbers of perpendicular-orientated SaOS-2 cells were also seen with longer durations of dcEF stimulation (Fig. 1H), but this effect became significant only after 3 h of exposure. In both cell types, the change in cell orientation became significant in a dcEF of 2 V/cm (21±2%, Mean±standard deviation) and highly increased at 5 V/cm (52±1.6%, Mean±standard deviation) (Fig. 1I and J). Overall, reorientation of the cells increased proportionally with both duration (Fig. 1G and H, n_cell_ = 1200–1500.) and strength (Fig. 1I and J, n_cell_ = 1200–1500) of the dcEF. This experiment was done in the presence of Ca^2+^.

### DcEF-induced directional migration is Ca^2+^-dependent

**Figure 2:**
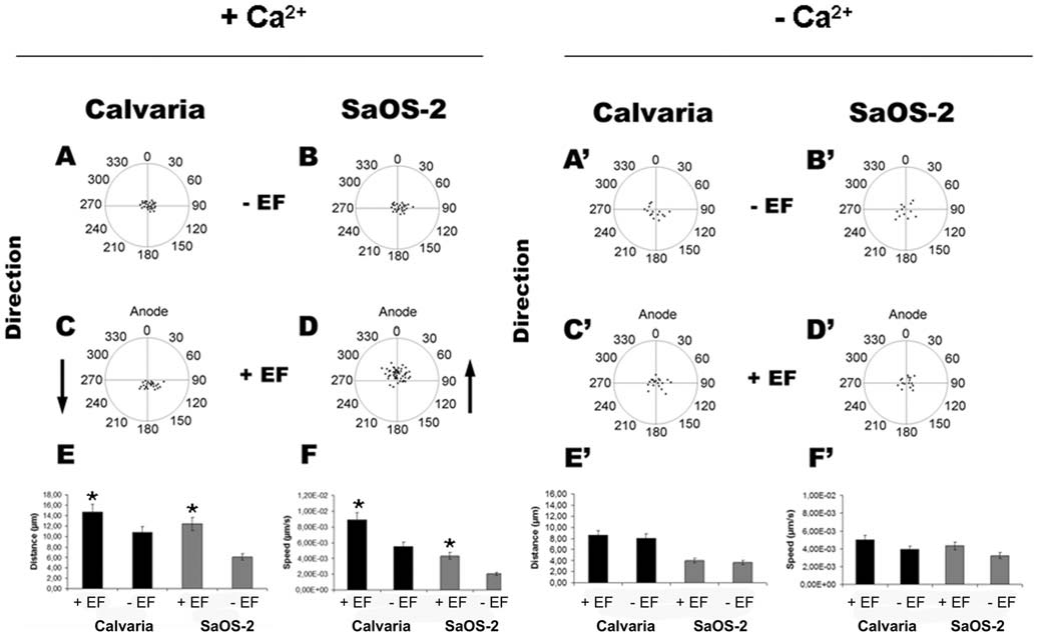
DcEF (5 V/cm, 3 h)-directed cell migration in the presence or absence of Ca^2+^ ions. Control calvaria and SaOS-2 cells migrate randomly either in the presence (A,B) or absence (A',B') of Ca^2+^ ions. Arrows indicate the opposite direction of calvaria (cathode, C) and SaOS-2 (anode, D) cells in an EF in the presence of Ca^2+^ ions. No directional migration is observed in the absence of Ca^2+^ ions (C',D'). Increased distance (µm) and speed (µm/sec) in EF-directed cell migration in the presence (E,F) but not in the absence of Ca^2+^ ions (E',F'). Means and standard deviations are shown. * indicates P<0.05. Data was collected from 17–22 cells from at least three independent experiments.

To determine if Ca^2+^ is important for dcEF-induced directed migration, we compared cell behavior in the presence and absence of Ca^2+^. In the presence of Ca^2+^, the two osteogenic cell types migrated in opposite directions when exposed to a dcEF, calvarial cells to the cathode (Fig. 2C, Movie S1, Fig. S1) and SaOS-2 cells to the anode (Fig. 2D, Movie S2, Fig. S2). Unexposed cells moved randomly (Fig. 2A and B). Without Ca^2+^, both SaOS-2 (Fig. 2D') and calvarial (Fig. 2C') osteoblasts did not show a preferred direction, similar to the control cells (Fig. 2A' and B'). The data showing the values of directedness in degree has given in the supporting information (Dataset S1). The presence of Ca^2+^ increased the net linear displacement from 6.08 µm to 12.42 µm for SaOS-2 cells and from 10.86 µm to 14.72 µm for calvarial cells exposed to a 5 V/cm dcEF for 5 h (Fig. 2E). Net linear displacement was defined by the starting and end points connected by a straight line. Migration speeds calculated from the original trajectories were 20 µm/hr (calvarial) and 7.3 µm/hr (SaOS-2) for unexposed cells, and 32.1 µm/hr (calvarial) and 15.5 µm/hr (SaOS-2) for dcEF-exposed cells (Fig. 2F). DcEFs triggered an increase in distance and speed of calvarial osteoblast movement by 36±3% and 61±5.2%, while SaOS-2 cells increased their distance and speed by 104±8% and 111±10.5% respectively. Without Ca^2+^, dcEFs did not cause distinguishable differences in migration speed and distance compared to the control cells (Fig. 2E' and F'). Mean values were evaluated from at least 3 individual experiments for each case (n_cells_ = 17–22). In total, this data suggests that dcEFs guide osteoblast migration via a Ca^2+^-dependent mechanism.

### DcEF-induced redistribution of adhesion structures, elongation and de novo formation of membrane protrusions is Ca^2+^-dependent

**Figure 3:**
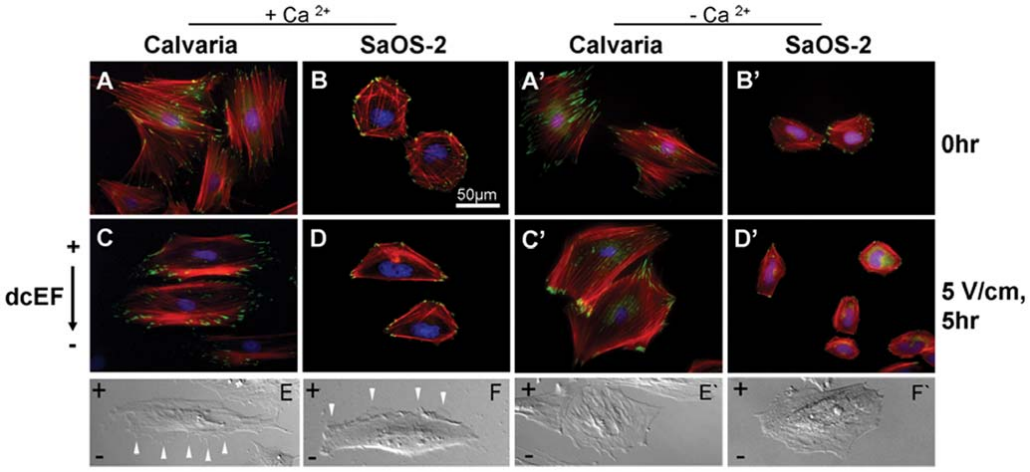
Redistribution of adhesion proteins (vinculin and actin) and de novo formation of membrane protrusions during dcEF (5 V/cm, 5 h) exposure. Fluorescent images of calvaria and SaOS-2 cells stained with phalloidin. TRITC, vinculin and DAPI mark actin filaments, focal contacts and nuclei, respectively before (A,B) and after (C,D) dcEF exposure in the presence, (A',B') or (C',D') absence of Ca^2+^ ions. DIC micrographs of elongated calvaria and SaOS-2 cells with newly formed membrane protrusions (white arrows) on their leading edges in presence of Ca^2+^ ions (E,F) or cells with randomly distributed protrusions absence of Ca^2+^ (E',F').

To further understand the Ca^2+^-dependent mechanism by which dcEFs direct migration, we looked at indicators of morphological rearrangement. Control cells (Fig. 3A and B) displayed a relatively homogenous distribution of focal adhesions, as marked by vinculin, and randomly arranged actin filaments. In the presence of Ca^2+^, dcEF-exposed SaOS-2 and calvarial cells had concentrations of vinculin spots near the cell edges or in the direction of movement (Fig. 3C and D). Parallel organized filamentous actin and new membrane protrusions (lamellipodia or filopodia) were also more noticeable at the leading edge of both cell types after dcEF exposure (Fig. 3C–F). When Ca^2+^ ions were depleted from the media, none of these changes were observed (Fig. 3A'–F'). This indicates that Ca^2+^ is required for the redistribution of focal adhesions and the actin cytoskeleton that occurs as these osteoblasts undergo morphological change during migration.

### Strong dcEFs elevate [Ca^2+^]_i_ by involving VGCCs

**Figure 4:**
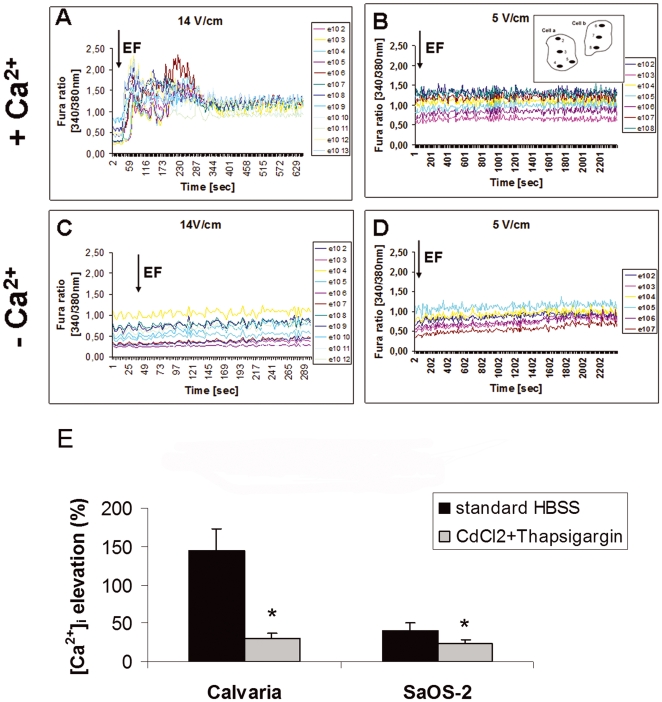
Fura-2AM kinetics showing [Ca^2+^]_i_ elevation during dcEF-induced migration is dependent on Ca^2+^ and EF strength, and involves VGCCs. The dcEF (black arrows) was applied 30 seconds after starting the time-lapse recordings. Elevated and steady state Ca^2+^ levels of calvaria cells exposed to strong (14 V/cm) or weak (5 V/cm) of dcEF in the presence of Ca^2+^ (A,B). In the absence of Ca^2+^, both strong (C) and weak (D) dcEF failed to stimulate intracellular Ca^2+^. A scheme of two cells with marked ROIs is shown for B. Lines in each graphic correspond to different groups of ROIs from different cells and are listed in the legends. 20–23 cells with similar kinetics were scored from 3–6 individual experiments for each condition. (E) Inhibition of dcEF-induced [Ca^2+^]_i_ elevation by VGCC blocker CdCl_2_ in both calvarial and SaOS-2 osteoblast-like cells. 8 cells from 5 individual experiments were analyzed for each condition. Means and standard deviations are shown. * indicates P<0.05 vs. the elevation in the normal conditions without the blocker.

To address whether dcEF-induced cell migration involves regulating intracellular calcium levels, we monitored [Ca^2+^]_i_ dynamics in response to different strength dcEFs. After the onset of exposure to a 14 V/cm dcEF in the presence of Ca^2+^, calvarial osteoblasts (Fig. 4A) had a detectable [Ca^2+^]_i_ increase at an average of 5.87 seconds (n_cell_ = 23, n_ROI_ = 90, n_exp_ = 10), while SaOS-2 cells took 11.73 seconds (n_cell_ = 22, n_ROI_ = 73, n_exp_ = 6). The amplitude of the elevation calculated from the baseline was 143.82±29.5% (Mean±standard deviation, n_cell_ = 23, n_ROI_ = 90, n_exp_ = 10) for calvarial osteoblasts and 41.16±9.96% (Mean±standard deviation, n_cell_ = 22, n_ROI_ = 73, n_exp_ = 6) for SaOS-2 cells. In contrast, intracellular Ca^2+^ levels were kept at steady state under low strength dcEF (5 V/cm) exposure under the same conditions (Fig. 4B, n_exp_ = 4). Without Ca^2+^, cells responded neither to a 5 V/cm (Fig. 4D, n_exp_ = 4) nor a 14 V/cm (Fig. 4C, n_exp_ = 4) dcEF. Thus, increased [Ca^2+^]_i_ could serve as a rapid response mechanism to regulate cell migration in response to strong dcEFs.

Depletion of intracellular Ca^2+^ stores using 10 µM thapsigargin and the subsequent incubation of cells with 50 µM of the VGCC blocker cadmium chloride (CdCl_2_) reduced the peak magnitude of [Ca^2+^]_i_ by 78.03% (n_cell_ = 8) in calvarial cells and by 43.44% (n_cell_ = 8) in SaOS-2 cells (Fig. 4E). Additionally, the SaOS-2 [Ca^2+^]_i_ elevation in response to the applied dcEF was delayed by 2±0.3 min (Mean±standard deviation, n_cell_ = 8, Fig. S3). This suggests that dcEFs regulate [Ca^2+^]_i_ via VGCCs.

### Strong dcEF induced [Ca^2+^]_i_ elevation on opposite sides in Calvarial and SaOS-2 cells

**Figure 5:**
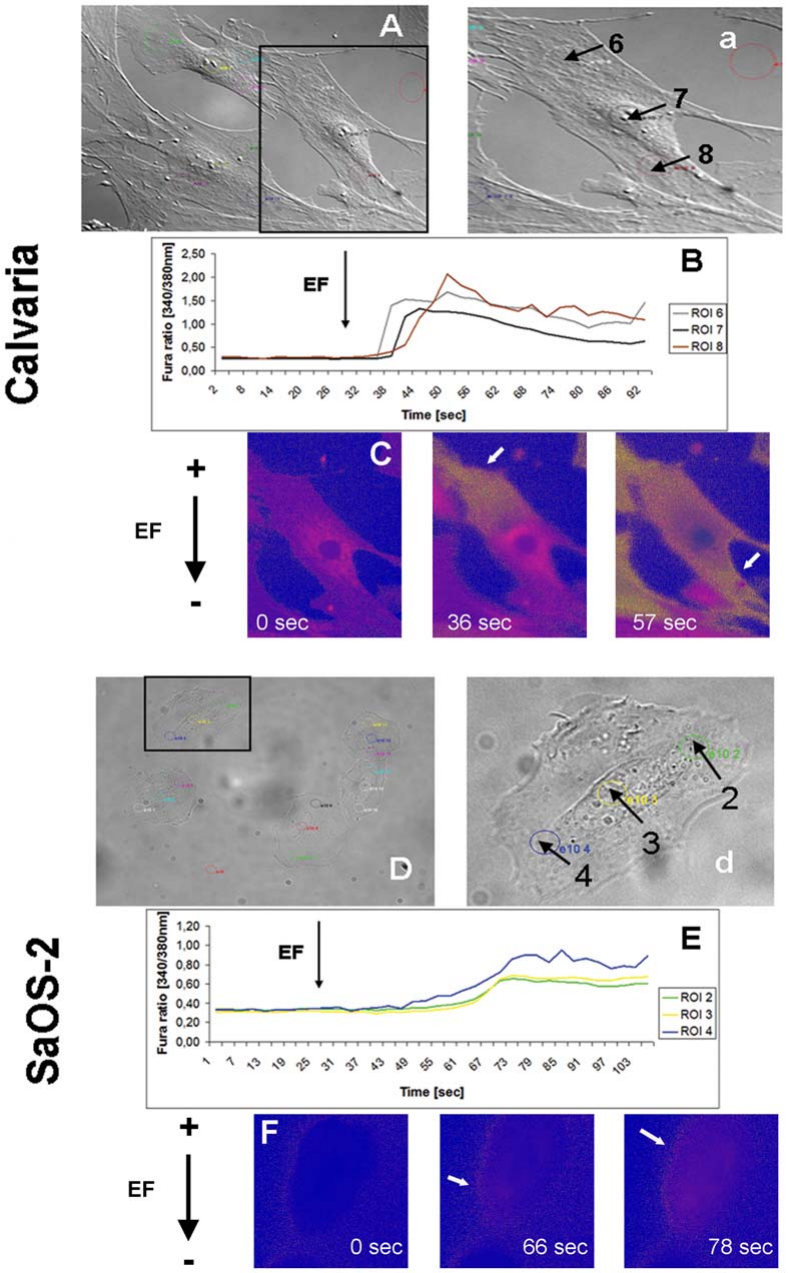
Local and opposite Ca^2+^ initials in Fura-loaded calvaria and SaOS-2 cells exposed to strong dcEF (14 V/cm). (A,a) and (D,d) DIC images of calvaria and SaOS-2 cells with ROIs within colored circles. (B) One-cell Fura kinetics representing the sequential elevation of [Ca^2+^]_i_ first at anode-facing side (ROI 6, grey circle) and its propagation through the cell (ROI 7, black circle) to the cathode-facing side (ROI 8, dark red circle) of a calvaria cell. (E) The same pattern but opposite direction in a SaOS-2 cell. (C,F) False color time-lapse frames showing the contrary initiation and propagation (white arrows) of [Ca^2+^]_i_ elevation in calvaria and in SaOS-cells. 15–17 cells from a total of 22–23 were scored from 6–10 independent experiments.

To determine if there is a relationship between local calcium kinetics and directional sensing, we visualized [Ca^2+^]_i_ at the cellular level in both calvarial (Fig. 5A and a) and SaOS-2 cells (Fig. 5D and d) after exposure to a 14 V/cm dcEF. Different cellular regions showed Ca^2+^ elevation at different time points (Fig. 5B, 17 cells scored from a total of 23, n_exp_ = 10). A local rise in [Ca^2+^]_i_ appeared first at the anode-facing side of the calvarial cells, then spread until it reached the opposite, cathode-facing, extremity (Fig. 5C, Movie S3). Unlike calvarial osteoblasts, Ca^2+^ elevation in SaOS-2 initiated first at the cathode-facing end, then diffused through the cytoplasm until it reached to the anode-facing end of the cell (Fig. 5D–F, Movie S4, 15 cells scored from a total of 22, n_exp_ = 6). Thus dcEFs trigger different patterns of intracellular Ca^2+^ elevation in osteogenic cell types that migrate in opposite directions.

### DcEFs induce cells to initially contract and relocate opposite to migration direction

**Figure 6:**
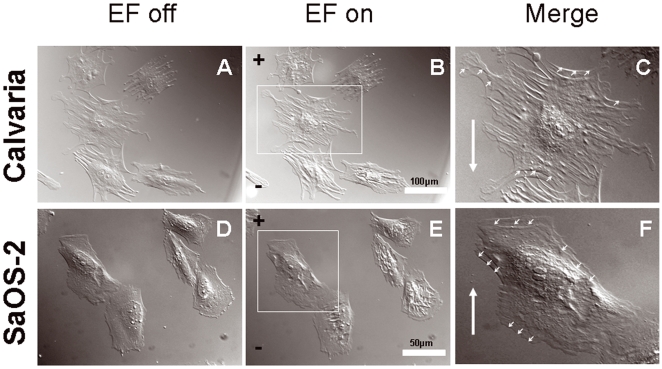
DIC time-lapse frames showing opposing contraction forces induced by dcEFs. (A,D) Calvaria osteoblasts and SaOS-2 cells before dcEF exposure. (B,E) Calvaria and SaOS-2 cells with contracted cell bodies 1 min after switching the dcEF (10 V/cm applied here) on. (C,F) Merged images of a single calvaria and SaOS-2 cell, marked with a white rectangle in B and in E, representing contraction and positional shift (small white arrows on the cell periphery) towards anode and cathode, respectively. Big white arrow in C and in F indicates the direction of calvaria and SaOS-2 cell migration towards cathode and anode, respectively. n_cell_ = 17–21.

We noticed that Calvarial (n = 17) and SaOS-2 (n = 21) cells (Fig. 6A and D) underwent a significant contraction within 1 minute of exposure to dcEF (Figs. 6B and E). Here, dcEF strength of 10 V/cm was chosen as representative as this effect was also observed with other strengths of dcEF (1–14 V/cm). Calvarial osteoblasts contracted towards the anode (Fig. 6C, Movie S5) and SaOS-2 cells towards the cathode (Fig. 6F, Movie S6). Interestingly, in both cell types the relocation (positional shift) caused by the contraction was always opposite to the preferred migratory direction. This suggests that there is a refractory period during which the cells mechanically resist dcEF forces.

## Discussion

**Figure 7:**
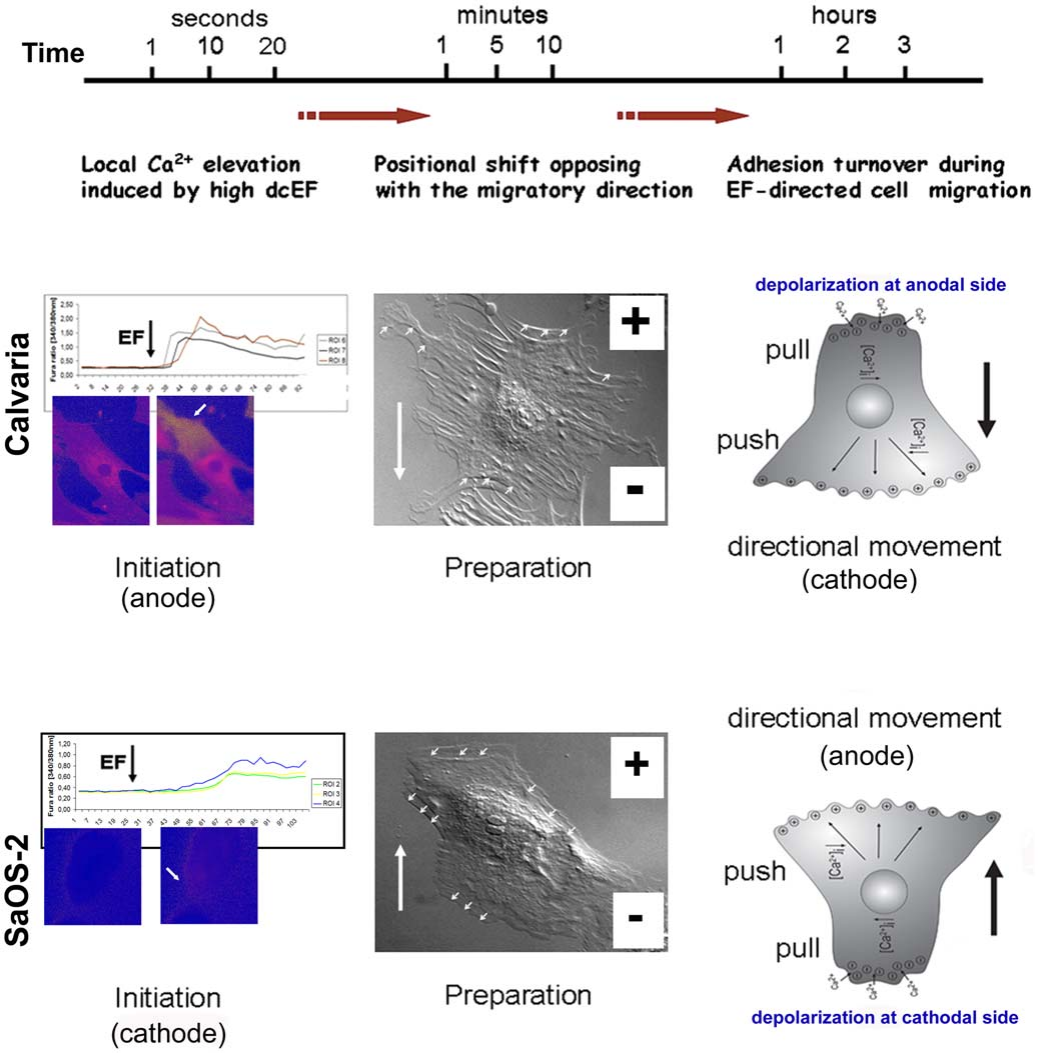
Flow diagram showing dcEF-induced early and late events in a single calvarial or SaOS-2 osteoblast cell. Strong dcEF triggers a local elevation in intracellular calcium (in seconds) on the anode-facing cell side followed by a shift (in minutes) opposite to migration direction, towards the anode, and finally, directed migration (in hours) towards cathode in a calvarial cell (upper panel). Similar mechanism, but in an opposite direction, translates the local increase in [Ca^2+^]_i_ at cathode facing side of a SaOS-2 cell into the movement towards anode (lower panel).

Here we describe the initial events during dcEF-induced directional cell migration in osteoblasts and show that Ca^2+^is essential (Fig. 7). This is in contrast to other cell types reported to be regulated independent of Ca^2+^, such as NIH 3T3 and SV101 murine fibroblastic cell lines [20]. In particular, we find that dcEFs require Ca^2+^ to induce morphological changes that enable migration. A Ca^2+^-dependent mechanism has previously been suggested to explain dcEF-induced cell shape changes, orientation and displacement of mouse embryo fibroblasts [21]. Inhibition of lamellipod formation and cell locomotion has also been reported in dcEF-guided fish keratinocytes exposed to Ca^2+^ channel antagonists [22].

Our data suggests that regulation of intracellular Ca^2+^ levels is a key for dcEF induction of cell migration responses. Notably, directed motility and intracellular Ca^2+^ kinetics in osteoblast-like cells can be altered by different strength dcEFs. This effect might be related to VGCC activation occurring only at strong dcEFs in osteoblasts, as a similar situation has been described for hFOB 1.19 osteoblasts [23]. Inhibiting VGCCs using CdCl_2_ significantly reduced [Ca^2+^]_i_ peak magnitude in calvarial and SaOS-2 cells. Thus, VGCCs may contribute to Ca^2+^ entry across the plasma membrane at strong dcEFs.

We show for the first time here that dcEFs trigger different patterns of intracellular Ca^2+^ elevation in osteogenic cell types that migrate in opposite directions. In combination with our other data, this leads us to propose a model of how dcEFs affect local calcium kinetics to guide migration of a SaOS-2 cell. The resting potential of osteoblast-like cells is around −20 to −30 mV [24], [25]. A dcEF of 10 V/cm will affect a 50 µm diameter (in average) SaOS-2 cell by hyperpolarizing the membrane facing the anode by approximately 50 mV and depolarizing the cathodal side by the same amount [26]. Increasing [Ca^2+^]_i_ can activate myosin light chain kinase, which in turn triggers actin-activated myosin ATPase, a major regulator of cell contraction [27]. As a consequence, the cathodal side will contract and propel the SaOS-2 cell towards the anode. A cathode-facing membrane depolarization may open VGCCs and the following Ca^2+^ influx will enhance the local [Ca^2+^]_i_, causing cell migration towards the anode. Stretch-activated cation channels, including calcium-related ones involved in mechanotransduction [28], are present in osteoblasts and are crucial for maintaining bone density [29]. Thus, stretch-activated calcium channels may especially contribute to the local [Ca^2+^]_i_ increase due to the mechanical strain induced by the EF, and cause a depolarization-activated calcium response [30] at the rear-end (cathode) of the SaOS-2 cell. Similar mechanism, but in an opposite direction, will translate the local increase in [Ca^2+^]_i_ at the anode facing side of a calvarial cell into movement towards the cathode. Nevertheless, how and why [Ca^2+^]_i_ rises on the anodal side for calvarial and cathodal side in SaOS-2 cells is still an issue to be discussed. One possible basis for this opposite effect could be the status of VGCCs in the two cell lines used. Because of CdCl_2_ being a non-specific VGCC inhibitor, its use can only address the VGCCs in general, but can not reveal VGCC subtypes involve in [Ca^2+^]_i_ rise in two cell lines exposed to strong dcEF. Additionally, Ca^2+^-permeant mechanosensitive channels might be secondary modulators to VGCCs contributing to the differential directionality of response.

However, it is likely that another mechanism keeps [Ca^2+^]_i_ in a steady state during weak (5 V/cm) dcEF-directed motility. Hereby, Ca^2+^ ions, but not necessarily local Ca^2+^ elevations, also seem to be involved in directed motility since the morphology and preferred migration direction of the cells was disrupted when Ca^2+^ was depleted from the media during the exposure to weak dcEF. A mechanism involving the local activation of some other channels/proteins might modulate directed cell migration in weak dcEFs.

Interestingly, we observed a rapid contraction and a slight relocation with a preferred directionality within the first minutes post-application of dcEFs (1–14 V/cm) in both calvarial and SaOS-2 cells. Each cell type showed a positional shift that was opposite to its preferred migrational direction, a behavior that we term the refractory period. During this time it is possible that the cells mechanically resist the dcEF forces until they reach the appropriate orientation. Considering that both the morphological reorganization and the positional shift noted here have preferred directions, they may be predetermining the migration direction and hence, required for electrically-guided cell migration. We suggest that the contraction and the shifting may also be occurring in response to the local calcium elevations at rear-end of the cells since both events refer to the same cell side with regard to the migration direction for the given cell type.

Cell migration with a preferred directionality may involve the local activation of various charged receptors or ion channels that subsequently contribute to migratory processes at the leading edge of the cell [7]. Moreover, as many molecules have charges on them, it is likely that electrical stimulation directly induces the activity of e.g. membrane proteins participating in ion transport or other molecules acting as a voltage sensor such as ciVSP, a recently identified voltage sensing phosphatase [31]. Therefore, investigating the roles of such proteins and associated mechanisms for translating electrical signals into cell biological events producing guided cell migration is our future aim.

In conclusion, the findings here provide insight into the involvement of cell- and dcEF-specific cues in guided migration. Our overview of the time scale of electrically-directed migration in osteoblast-like cells, presented for the first time here, helps to understand how physiological electrical signals can initiate directional sensing in the cells. This will contribute to our understanding of important biological events in which guided migration is crucial, such as bone remodeling.

## Materials and Methods

### Osteoblast cell culture

Primary osteoblastic cells were isolated from fetal rat calvaria as previously described [32]. Cultures were maintained in DMEM/Ham's F12 medium (Gibco BRL, Karlsruhe, Germany) containing 12% fetal calf serum, 2.3 mM Mg^2+^, 100 µg/mL penicillin, 100 µg/ml streptomycin sulfate and 1.25% L-glutamine at 37°C and humidified 5% CO_2_. Passages between 5 and 7 were taken for experiments. Human osteosarcoma cells (SaOS-2), non-transformed cells with osteoblastic properties, were obtained from the American Type Culture Collection (ATCC HTB 85) and cultured in McCoy's 5A medium (Gibco BRL, Karlsruhe, Germany) containing 15% fetal calf serum and 1.25% L-glutamine. SaOS-2 cells were used up to passage number 50 since they are suspected to loose their osteoblast-phenotypic features in later passages [33]. 100 µL of 20000 cells/mL was seeded into the channel of an ibiTreat - μ Slide I (Ibidi, Munich, Germany) for EF applications.

### Application of dcEFs

Direct current provided by an electrophoresis power supply (Amersham Pharmacia Biotech, Freiburg, Germany) was applied to the cells through two platinum electrodes (0.2 mm diameter, Agar scientific, Essex, UK) immersed in 0.9% NaCl-filled beakers that were connected to the media-filled reservoirs of the μ Slide by two approximately 20 cm long agar bridges (2% agar in PBS). HEPES buffered salt solution (HBSS; 10 mM HEPES, 135 mM NaCl, 1.2 mM CaCl_2_, 1.2 mM MgCl_2_, 5 mM KCl and 0.8% D-Glucose, pH 7.4) or cell culture media was used for ion imaging or migration assays, respectively. Agar bridges were used to avoid contact between electrode products and the cells [2]. Field strengths were measured during the experiment by a voltmeter (Voltcraft® Meβtechnik, Hirschau, Germany). Cells were exposed to the different dcEF strengths ranging from 1 to 14 V/cm for 5 min up to 5 h at 37°C in a chamber (Solent Scientific, Segensworth UK) covering the whole inverted microscope or in the incubator. Same conditions were used for controls but without application of a dcEF.

### Quantification of cell orientation and migration

Cells were monitored with an Olympus IX81 inverted microscope equipped with DIC components and an integrated vital microscopy chamber (Olympus, Hamburg, Germany). Cells with an angle between 70° and 110° to the EF vector were considered as perpendicular. A total of 1000–1500 cells were scored for each condition. To quantify cell migration, time-lapse DIC videos were processed in a detecting program, TrackIT. Diagrams indicating the direction of cell migration were evaluated in Microsoft Power Point from original cell trajectories detected by TrackIT. Graphics of speed and distance were prepared in Excel from the original data generated in TrackIT.

### Fluorescence labeling of actin, vinculin, and nuclei

Cells were washed once with PBS (pH 7.4), fixed in 4% formaldehyde for 5 min at room temperature (RT), permeabilized with 0.5% Triton X-100 for 6 min and then blocked with 1% bovine serum albumin (BSA) for 20 min. To detect focal contacts, the cells were incubated with a mouse anti-human vinculin (1∶20, Serotec, Martinsried, Germany) at 4°C overnight. After washing with PBS, the cells were incubated with a fluorescein-isothiocyanate (FITC)-coupled goat anti-mouse antibody (1∶100, Dianova, Hamburg, Germany) for 1 h at RT. To visualize the actin cytoskeleton, cells were incubated with tetramethylrhodamine-isothiocyanate (TRITC)-conjugated phalloidin (1∶20, Sigma-Aldrich, Munich, Germany) for 1 h at RT. Nuclei were stained with 4′,6-diamidino-2-phenylindole dihydrochloride, DAPI (1∶50, Sigma-Aldrich, Munich, Germany) for 5 min at RT. 2% Dabco-glycerin in PBS was used as mounting solution.

### Monitoring intracellular calcium

Fura-2 AM (Invitrogen, Karlsruhe, Germany), a Ca^2+^-specific vital dye, was used to record intracellular calcium levels. Cells were washed once with HBSS and then loaded with 3 µM Fura-2 AM, 0.05% Pluronic F-127 (Sigma-Aldrich, Munich, Germany) and 3% FCS in HBSS at 37°C for 15 min. After washing, cells were incubated further with HBSS containing 3% FCS for 10 min, then rinsed with HBSS and examined using an inverted microscope. Ca^2+^ free conditions were ensured by using 1.2 mM EGTA (Applichem, Darmstadt, Germany) and 10 µM Thapsigargin (Calbiochem, Darmstadt, Germany) to define the requirement of extracellular calcium ions and calcium ions released from the intracellular stores. For VGCC blocking studies, intracellular calcium stores were first depleted using Thapsigargin to ensure that an eventual elevation in [Ca^2+^]_i_ induced by the dcEFs was only due to the influx through the cell membrane. Afterwards the cells were further incubated with 50 µM CdCl_2_ (Sigma-Aldrich, Munich, Germany). A DcEF was applied approximately 30±1 sec after the start of the fluorescence recording during the experiments. The graphic legends with the number of series indicate the region of interests (ROIs) inserted in the cells in the given microscopic field. Data was recorded and analyzed using the imaging software Olympus CellˆR.

### Statistical analysis

Statistical calculations were performed either in Excel using Student's t-test or in SPSS using one-way analysis of variance (ANOVA) with Bonferroni test. P-values<0.05 were considered as significant (*). n_cell_, n_ROI_ and n_exp_, represent the total number of scored cells, total number of detected ROIs and the total number of independent experiments, respectively. Data were collected from at least three individual experiments.

## Supporting Information

Movie S1 DcEF-directed motility in calvarial osteoblasts. DIC time-lapse video showing dcEF-directed motility of calvarial osteoblasts exposed to 5 V/cm for 5 h. Calvarial osteoblasts elongate perpendicular to EF vector and form membrane protrusions towards cathode (−) during EF-induced directional migration.
(8.28 MB MOV)

Movie S2 DcEF-directed motility in SaOS-2 osteoblast-like cells. DIC time-lapse video showing dcEF-directed motility of SaOS-2 cells exposed to 5 V/cm for 5 h. SaOS-2 cells elongate perpendicular to EF vector and form membrane protrusions towards anode (+) during EF-induced directional migration.
(9.06 MB MOV)

Movie S3 DcEF-induced local Ca2+ elevation in calvarial osteoblasts. Time-lapse video showing EF-induced elevation of Ca2+ first at anode-facing side and its propagation through the cell to the cathode-facing side of calvarial osteoblasts loaded with Fura-2 AM.
(2.24 MB MOV)

Movie S4 DcEF-induced local Ca2+ elevation in SaOS-2 osteoblast-like cells. Time-lapse video showing EF-induced elevation of Ca2+ first at cathode-facing side and its propagation through the cell to the anode-facing side of SaOS-2 cells loaded with Fura-2 AM.
(1.64 MB MOV)

Movie S5 DcEF-induced positional shift in calvarial osteoblasts. DIC time-lapse video showing contraction and positional shift of calvaria osteoblasts towards anode (+). EF was switched on and off for 5 and 1 minutes, respectively during the experiment. The relocation (positional shift) caused by contraction is opposite to the preferred migration direction, which is towards cathode (−) for these cells.
(8.09 MB MOV)

Movie S6 DcEF-induced positional shift in SaOS-2 osteoblast-like cells. DIC time-lapse video showing contraction and positional shift of SaOS-2 cells towards cathode (−). EF was switched on and off for 5 and 1 minutes, respectively during the experiment. The relocation (positional shift) caused by the contraction is opposite to the preferred migratory direction, which is towards anode (+) for these cells.
(9.14 MB MOV)

Figure S1 Migration direction of calvarial osteoblasts exposed to dcEF. Original images showing directions, pathways and linear displacements of calvarial osteoblasts towards cathode (at 180°). Cells were exposed to 5 V/cm for 5 h. Data was generated using cell tracking program Olympus cellˆR-TrackIT.
(1.69 MB TIF)

Figure S2 Migration direction of SaOS-2 osteoblast-like cells exposed to dcEF. Original images showing directions, pathways and linear displacements of SaOS-2 cells towards anode (at 0°). Cells were exposed to 5 V/cm for 5 h. Data was generated using cell tracking program Olympus cellˆR-TrackIT.
(1.58 MB TIF)

Figure S3 Inhibition of dcEF-induced [Ca2+]i elevation by CdCl2. Graphics showing reduced [Ca2+]i elevation in response to CdCl2 recorded from calvarial and SaOS-2 osteoblast-like cells loaded with Fura-2AM. EF was applied 20 min after the onset of incubation with 50 µM CdCl2.
(0.71 MB TIF)

Dataset S1 Directedness of EF-guided osteoblast-like cells in the presence or absence of Ca2+ ions. Values between 90°–270° corresponds to the directions towards cathodal- and those between 0°–90° or 270°–360° towards anodal side. Ncell = 15–22 for each condition.
(0.02 MB XLS)

## References

[1] Trollinger DR, Isseroff RR, Nuccitelli R (2002). Calcium channel blockers inhibit galvanotaxis in human keratinocytes.. J Cell Physiol.

[2] Zhao M, Agius-Fernandez A, Forrester JV, McCaig CD (1996). Directed migration of corneal epithelial sheets in physiological electric fields.. Invest Ophthalmol Vis Sci.

[3] Bai H, McCaig CD, Forrester JV, Zhao M (2004). DC electric fields induce distinct preangiogenic responses in microvascular and macrovascular cells.. Arterioscler Thromb Vasc Biol.

[4] McCaig CD, Rajnicek AM, Song B, Zhao M (2005). Controlling cell behavior electrically: current views and future potential.. Physiol Rev.

[5] Funk RH, Monsees TK (2006). Effects of electromagnetic fields on cells: physiological and therapeutical approaches and molecular mechanisms of interaction. A review.. Cells Tissues Organs.

[6] Levin M (2007). Large-scale biophysics: ion flows and regeneration.. Trends Cell Biol.

[7] Funk RH, Monsees T, Ozkucur N (2009). Electromagnetic effects - From cell biology to medicine.. Prog Histochem Cytochem.

[8] Fang KS, Ionides E, Oster G, Nuccitelli R, Isseroff RR (1999). Epidermal growth factor receptor relocalization and kinase activity are necessary for directional migration of keratinocytes in DC electric fields.. J Cell Sci.

[9] Cho MR, Thatte HS, Lee RC, Golan DE (1996). Reorganization of microfilament structure induced by ac electric fields.. Faseb J.

[10] Zhao M, Song B, Pu J, Wada T, Reid B, Tai G (2006). Electrical signals control wound healing through phosphatidylinositol-3-OH kinase-gamma and PTEN.. Nature.

[11] Pinton P, Giorgi C, Siviero R, Zecchini E, Rizzuto R (2008). Calcium and apoptosis: ER-mitochondria Ca2+ transfer in the control of apoptosis.. Oncogene.

[12] Wayman GA, Lee YS, Tokumitsu H, Silva A, Soderling TR (2008). Calmodulin-kinases: modulators of neuronal development and plasticity.. Neuron.

[13] Roderick HL, Cook SJ (2008). Ca2+ signalling checkpoints in cancer: remodelling Ca2+ for cancer cell proliferation and survival.. Nat Rev Cancer.

[14] Titushkin IA, Rao VS, Cho MR (2004). Mode- and Cell-Type Dependend Calcium Responses Induced by Electrical Stimulus.. IEEE Trans Plasma Sci.

[15] Rodan SB, Imai Y, Thiede MA, Wesolowski G, Thompson D (1987). Characterization of a human osteosarcoma cell line (Saos-2) with osteoblastic properties.. Cancer Res.

[16] Ho ML, Chang JK, Chuang LY, Hsu HK, Wang GJ (1999). Characteristics of primary osteoblast culture derived from rat fetal calvaria.. Kaohsiung J Med Sci.

[17] Curtze S, Dembo M, Miron M, Jones DB (2004). Dynamic changes in traction forces with DC electric field in osteoblast-like cells.. J Cell Sci.

[18] Ferrier J, Ross SM, Kanehisa J, Aubin JE (1986). Osteoclasts and osteoblasts migrate in opposite directions in response to a constant electrical field.. J Cell Physiol.

[19] Binderman I, Somjen D, Shimshoni Z, Levy J, Fischler H (1985). Stimulation of skeletal-derived cell cultures by different electric field intensities is cell-specific.. Biochim Biophys Acta.

[20] Brown MJ, Loew LM (1994). Electric field-directed fibroblast locomotion involves cell surface molecular reorganization and is calcium independent.. J Cell Biol.

[21] Onuma EK, Hui SW (1988). Electric field-directed cell shape changes, displacement, and cytoskeletal reorganization are calcium dependent.. J Cell Biol.

[22] Cooper MS, Schliwa M (1986). Motility of cultured fish epidermal cells in the presence and absence of direct current electric fields.. J Cell Biol.

[23] Khatib L, Golan DE, Cho M (2004). Physiologic electrical stimulation provokes intracellular calcium increase mediated by phospholipase C activation in human osteoblasts.. Faseb J.

[24] Civitelli R, Reid IR, Halstead LR, Avioli LV, Hruska KA (1987). Membrane potential and cation content of osteoblast-like cells (UMR 106) assessed by fluorescent dyes.. J Cell Physiol.

[25] Ferrier J, Ward-Kesthely A, Homble F, Ross S (1987). Further analysis of spontaneous membrane potential activity and the hyperpolarizing response to parathyroid hormone in osteoblastlike cells.. J Cell Physiol.

[26] Erickson CA, Nuccitelli R (1984). Embryonic fibroblast motility and orientation can be influenced by physiological electric fields.. J Cell Biol.

[27] Stull JT, Lin PJ, Krueger JK, Trewhella J, Zhi G (1998). Myosin light chain kinase: functional domains and structural motifs.. Acta Physiol Scand.

[28] Liedert A, Kaspar D, Blakytny R, Claes L, Ignatius A (2006). Signal transduction pathways involved in mechanotransduction in bone cells.. Biochem Biophys Res Commun.

[29] Turner CH (2006). Bone strength: current concepts.. Ann N Y Acad Sci.

[30] Wiltink A, Van Duijn B, Weidema AF, De Vos A, van der Meer JM (1994). Differential depolarization-activated calcium responses in fetal and neonatal rat osteoblast-like cells.. Calcif Tissue Int.

[31] Murata Y, Iwasaki H, Sasaki M, Inaba K, Okamura Y (2005). Phosphoinositide phosphatase activity coupled to an intrinsic voltage sensor.. Nature.

[32] Monsees TK, Barth K, Tippelt S, Heidel K, Gorbunov A (2005). Effects of different titanium alloys and nanosize surface patterning on adhesion, differentiation, and orientation of osteoblast-like cells.. Cells Tissues Organs.

[33] Hausser HJ, Brenner RE (2005). Phenotypic instability of Saos-2 cells in long-term culture.. Biochem Biophys Res Commun.

