# Supplementary figures and images for: Local Calcium Elevation and Cell Elongation Initiate Guided Motility in Electrically Stimulated Osteoblast-Like Cells
Source: PLoS One. 2009 Jul 3;4(7):e6131. doi: 10.1371/journal.pone.0006131 (PMC2702840; doi:10.1371/journal.pone.0006131)

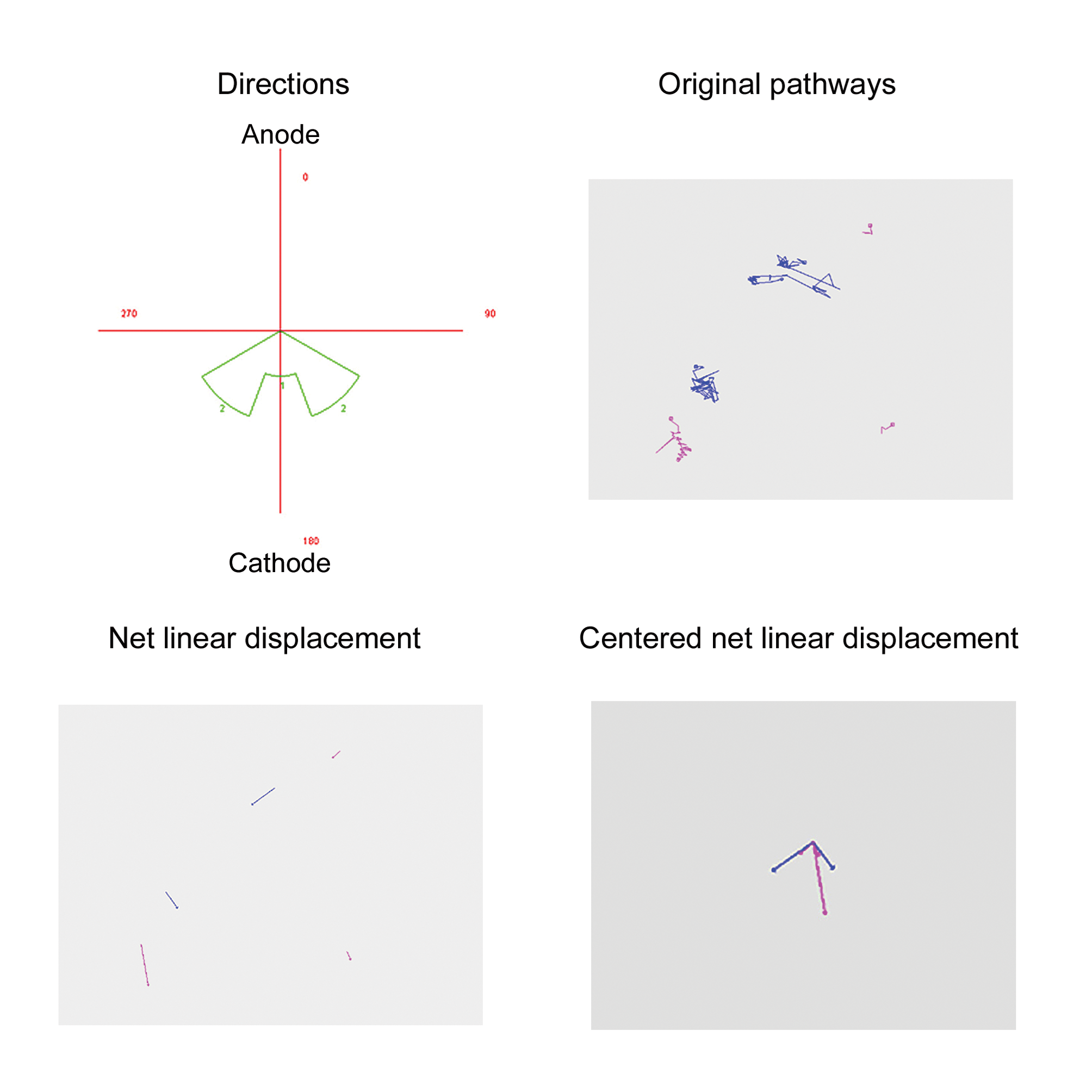

Supplement: Figure S1 — Migration direction of calvarial osteoblasts exposed to dcEF. Original images showing directions, pathways and linear displacements of calvarial osteoblasts towards cathode (at 180°). Cells were exposed to 5 V/cm for 5 h. Data was generated using cell tracking program Olympus cellˆR-TrackIT. (1.69 MB TIF) [file pone.0006131.s007.tif]

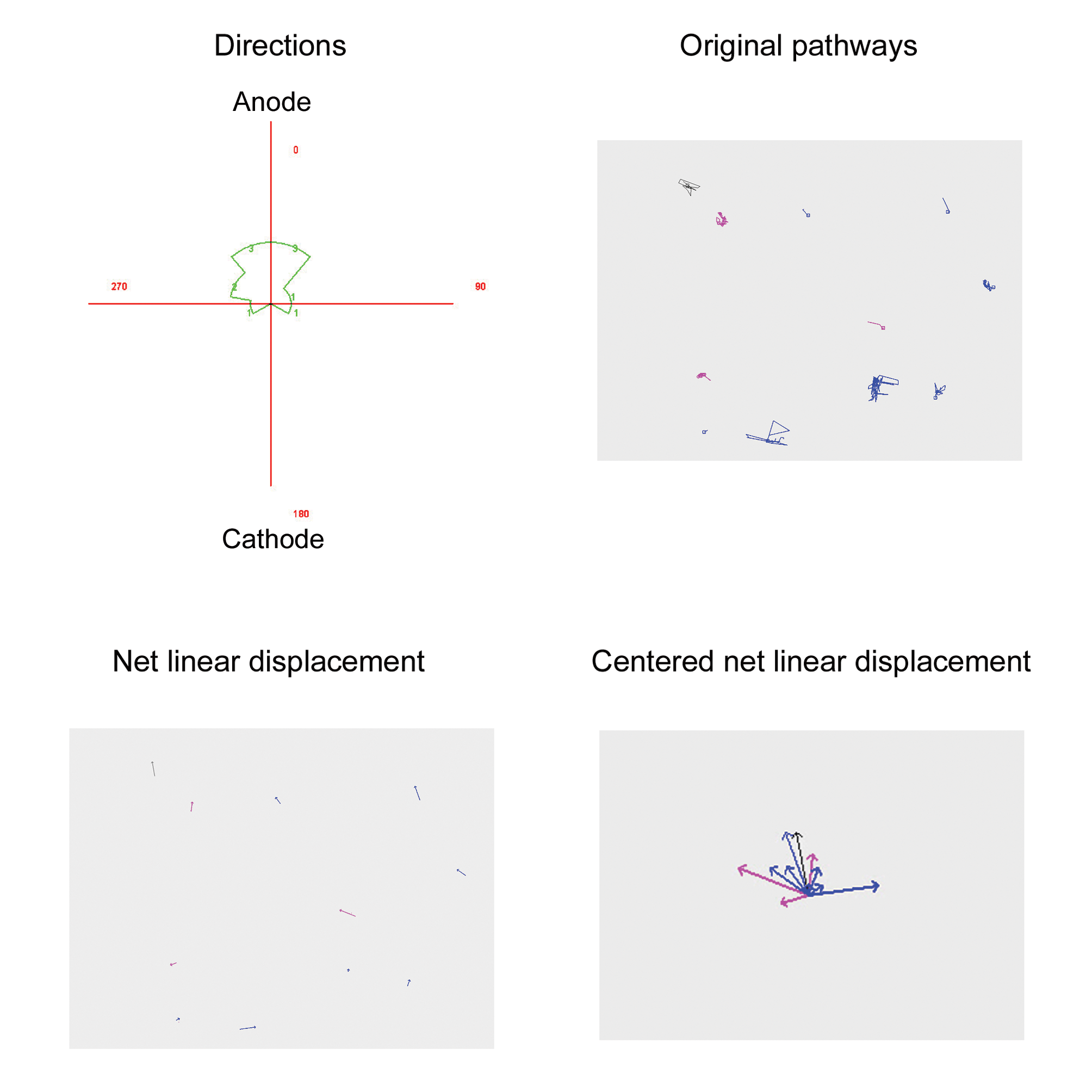

Supplement: Figure S2 — Migration direction of SaOS-2 osteoblast-like cells exposed to dcEF. Original images showing directions, pathways and linear displacements of SaOS-2 cells towards anode (at 0°). Cells were exposed to 5 V/cm for 5 h. Data was generated using cell tracking program Olympus cellˆR-TrackIT. (1.58 MB TIF) [file pone.0006131.s008.tif]

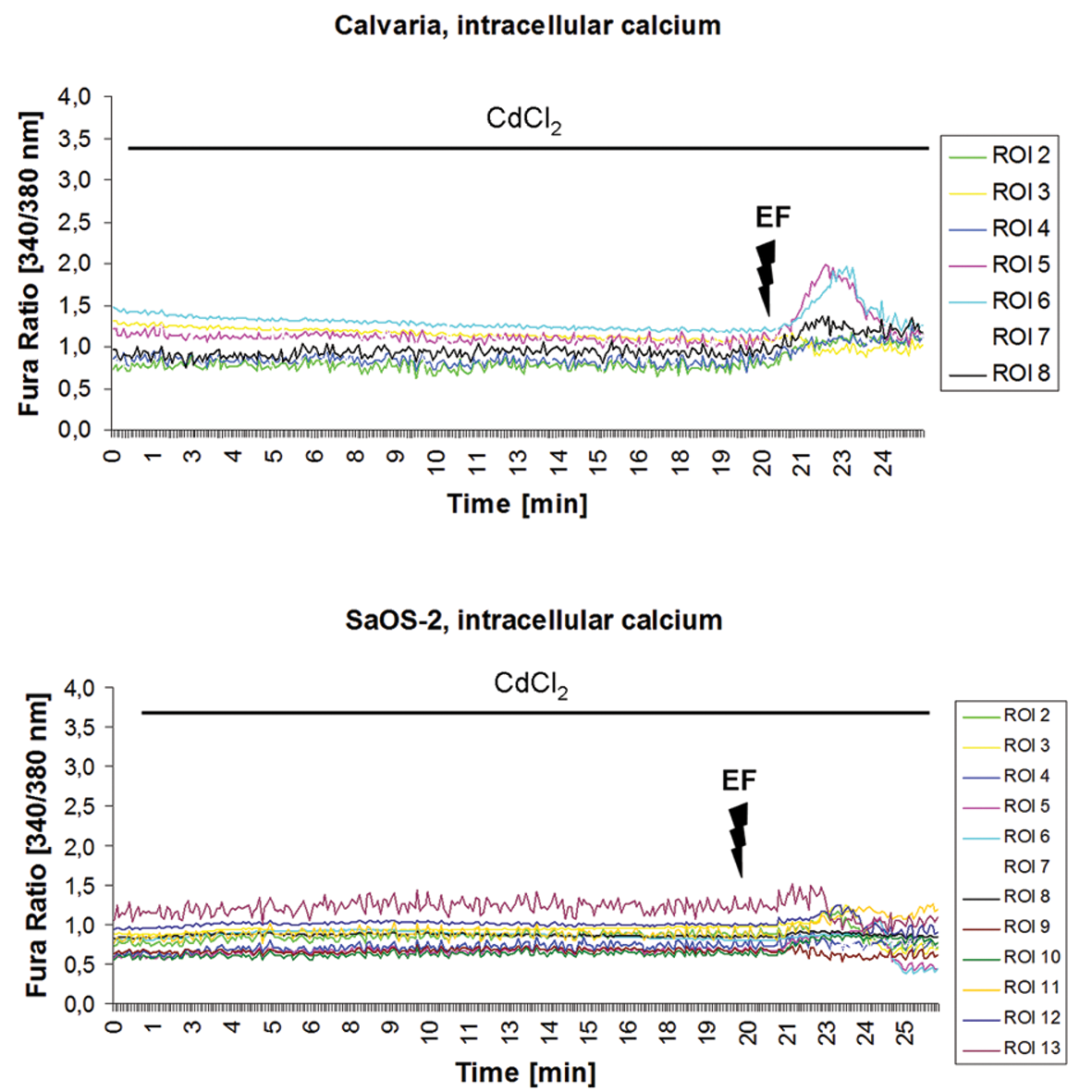

Supplement: Figure S3 — Inhibition of dcEF-induced [Ca2+]i elevation by CdCl2. Graphics showing reduced [Ca2+]i elevation in response to CdCl2 recorded from calvarial and SaOS-2 osteoblast-like cells loaded with Fura-2AM. EF was applied 20 min after the onset of incubation with 50 µM CdCl2. (0.71 MB TIF) [file pone.0006131.s009.tif]
